# Sensitive Electrochemical Biosensor for Rapid Screening of Tumor Biomarker TP53 Gene Mutation Hotspot

**DOI:** 10.3390/bios12080658

**Published:** 2022-08-19

**Authors:** Pengcheng Sun, Kai Niu, Haiying Du, Ruixin Li, Jiping Chen, Xianbo Lu

**Affiliations:** 1 College of Mechanical and Electronic Engineering, Dalian Minzu University, Dalian 116600, China; 2 CAS Key Laboratory of Separation Science for Analytical Chemistry, Dalian Institute of Chemical Physics, Chinese Academy of Sciences, 457 Zhongshan Road, Dalian 116023, China; 3 University of the Chinese Academy of Sciences, Beijing 100049, China

**Keywords:** biosensor, cancer biomarker, electrochemical, tumor suppressor gene, hairpin deoxyribonucleic acid, single nucleotide polymorphism

## Abstract

Rapid and sensitive detection of cancer biomarkers is crucial for cancer screening, early detection, and improving patient survival rate. The present study proposes an electrochemical gene-sensor capable of detecting tumor related TP53 gene mutation hotspots by self-assembly of sulfhydryl ended hairpin DNA probes tagged with methylene blue (MB) onto a gold electrode. By performing a hybridization reaction with the target DNA sequence, the gene-sensor can rearrange the probe’s structure, resulting in significant electrochemical signal differences by differential pulse voltammetry. When the DNA biosensor is hybridized with 1 μM target DNA, the peak current response signal can decrease more than 60%, displaying high sensitivity and specificity for the TP53 gene. The biosensor achieved rapid and sensitive detection of the TP53 gene with a detection limit of 10 nmol L^−1^, and showed good specific recognition ability for single nucleotide polymorphism (SNP) and base sequence mismatches in the TP53 gene affecting residue 248 of the P53 protein. Moreover, the biosensor demonstrated good reproducibility, repeatability, operational stability, and anti-interference ability for target DNA molecule in the complex system of 50% fetal bovine serum. The proposed biosensor provides a powerful tool for the sensitive and specific detection of TP53 gene mutation hotspot sequences and could be used in clinical samples for early diagnosis and detection of cancer.

## 1. Introduction

Despite advancements in medical care, cancer continues to be the most common cause of death. The key to cancer treatment lies in early detection, diagnosis, and treatment [1]. Currently, early detection of cancer is accomplished by the use of blood chip detection [2], gene detection [3], nano detection [4], positron emission tomography (PET) [5], computed tomography (CT) [6], and other approaches [7]. However, these approaches are highly limited in terms of early cancer detection due to their low sensitivity, high cost, and potential for physical or chemical harm [8]. Additionally, while enzyme-linked immunosorbent assay (ELISA) and polymerase chain reaction (PCR)-based technologies have high sensitivity and are less invasive, they require long detection times and involve high costs for operation [9,10,11]. As a result, creating analytical tools for the early detection of cancer is critical for medical diagnosis.

TP53 is a gene that acts as a tumor suppressor. This gene is mutated in more than 50% of all cancers [12,13] such as liver cancer [14], breast cancer [15], esophageal cancer [16], and lung cancer [17]. The majority of TP53 mutations in human tumors occur in the highly conserved region, with the highest mutation rates occurring at R175, R248, R249, R273, and R282 [18]. Due to its altered spatial conformation, TP53 loses its ability to regulate cell growth, apoptosis, and DNA repair, and ultimately transitions from a tumor suppressor to an oncogene [19]. As a result of the close association between the TP53 gene and cancer, conditions for early detection of cancer are created.

Over the last two decades, the importance of precise early illness detection and the development of personalized therapy have grown. In order to further improve the sensitivity and specificity of cancer marker detection, several novel analytical strategies such as electrochemical methods and electrochemiluminescence methods have been developed [20,21]. Thanks to its short detection time, distinctive hybridization specificity, and miniaturization potential, DNA-based electrochemical biosensors have attracted broad scientific and clinical interest recently [22]. Using electrochemical DNA biosensors based on the hybridization of DNA, specific target sequences can be subsequently measured, wherein a complementary capture probe recognizes the target DNA sequence [23]. The mass of the single-stranded DNA probe monolayer represents a major concern for building sensitive electrochemical biosensors, since the accessibility of target molecules is determined by the homogeneity, density, and upright conformation of ssDNA probes on the electrode surface [24,25]. The signal amplifier, the second component, converts minor changes in the interface into a detectable electrochemical signal [26]. Electrochemical DNA sensors have improved their detection performance when the single-stranded capture probe undergoes structural change from coiled to double-stranded DNA [23,27]. Concurrently, electrochemical DNA sensors provide improved selectivity for identifying tumor-related key genetic indicators such as single nucleotide polymorphism (SNP). The relatively poor selectivity of linear DNA probes for SNP is a typical problem in mutation analysis since the difference in melting temperatures between totally matched and SNP-containing hybrids can be as tiny as 1–2 °C [28]. If the mutation occurs within the loop area, the molecular beacon technique enhances this difference, resulting in greater discrimination between intact and mutant sequences [29]. Therefore, early diagnosis of patients with sensitive and accurate electrochemical gene-sensors may significantly improve patient survival rates [30,31]. Although TP53 gene mutation detection plays an important role in the early diagnosis of cancer, related electrochemical sensors have rarely been reported.

In this study, we constructed a simple and reliable gene-sensor based on an electronic DNA hairpin molecular beacon. Using the high-frequency mutation site R248 of the TP53 gene and its association with cancer, a hairpin probe was functionalized by modification of sulfhydryl and methylene blue (MB). The DNA biosensor with high sensitivity for SNP was designed to be able to effectively distinguish between intact, SNP-containing cancer biomarkers and three-base mismatch sequences, which is of great importance in early cancer screening.

## 2. Experimental Section

### 2.1. Chemicals and Reagents

6-Mercapto-1-hexanol (MCH) was obtained from J&K Chemical Ltd. (Beijing, China). Sodium chloride (NaCl), sulfuric acid (H_2_SO_4_), potassium dihydrogen phosphate (KH_2_PO_4_), dibasic sodium phosphate (Na_2_HPO_4_), potassium hexacyanoferrate (III) (K_3_Fe(CN)_6_), and potassium hexacyanoferrate (II) trihydrate (K_4_Fe(CN)_6_·3H_2_O) were obtained from Sigma (St. Louis, MO, USA). A Milli-Q purification system (Millipore, Burlington, MA, USA) with a resistivity of 18 MΩ·cm was used to obtain ultrapure water. Tris (2-carboxyethyl) phosphine hydrochloride (TCEP), tris-EDTA buffer solution (TE buffer) and the oligonucleotide sequences (Table S1 in supporting information) were synthesized by Sangon Biotech (Shanghai, China) Co., Ltd.

### 2.2. Preparation and Immobilization of DNA Sensors

Polycrystalline gold electrodes (2 mm diameter) were used to immobilize the SL-DNA probe. Polycrystalline gold electrodes were washed in a piranha solution (H_2_O_2_:H_2_SO_4_ = 1:3, *v/v*) for 15 min before being polished with 1.0, 0.3, and 0.05 m alumina slurry. Following rinsing, the electrodes were cleaned ultrasonically three times with ethanol followed by Milli-Q water. They were then electrochemically activated in 0.5 mol L^−1^ H_2_SO_4_ by potential scanning between 0 and 1.7 V until a repeatable CV was achieved, after which they were thoroughly washed with Milli-Q water and dried under a nitrogen stream [32,33].

**Figure 1:**
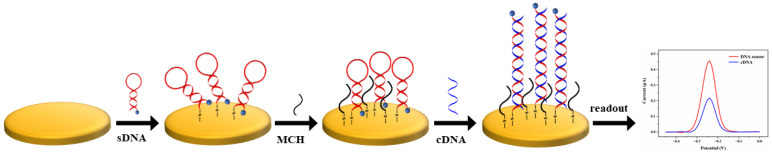
Schematic diagram of the fabrication and electrochemical response of the ssDNA modified gold electrode.

A gold-thiol bond was used to secure the ssDNA probe to the electrode surface. The disulfide bonds of the ssDNA probe were reduced with 10 mM TCEP by vibrating the solution at room temperature for 1 h. Following this, the solution was further diluted to obtain 0.01 μM, 0.1 μM, 1 μM, and 10 μM of the DNA probe in pH 7.4 phosphate-buffered saline (PBS). A total of 5 µL of this solution was drop cast onto the electrode and incubated at 4 °C for 16 h. The electrodes were submerged in PBS for 15 min and then dried with nitrogen stream. To avoid unintended adsorption on the electrode surface, the electrodes were dipped in 15 mL of 2 mM MCH in PBS and incubated for 1 h. Subsequently, the electrodes were immersed in PBS for 15 min and dried with nitrogen to obtain ssDNA-Au electrode (i.e., the geno-sensor). At the beginning of the hybridization reaction, 5 µL PBS solution containing cDNA were placed onto the ssDNA-Au electrodes and allowed to react at room temperature for 30 min. Subsequently, the electrodes were immersed in PBS for 15 min and dried with nitrogen to yield dsDNA-Au. The fabrication procedures for the biosensor are schematically depicted in Figure 1.

### 2.3. Electrochemical Measurements and Procedure

Cyclic voltammetry (CV), differential pulse voltammetry (DPV), and electrochemical impedance spectroscopy (EIS) experiments were performed in a three-electrode cell made of glass using a portable potentiostat, PalmSens4 (PalmSens4, Houten, The Netherlands) equipped with PS Trace 5.8 software (5.8.1704.0, PlamSens BV, Houten, The Netherlands). The prepared DNA sensor as the working electrode, an Ag/AgCl (3 M KCl) as the reference electrode, and a platinum wire as the auxiliary electrode. The assembly and hybridization reactions of the produced ssDNA-Au or dsDNA-Au were monitored using electrochemical scanning in PBS or [Fe(CN)_6_]^3−/^^4−^ solution. CV and DPV were performed on ssDNA-Au or dsDNA-Au from −0.5 to 0 V at a scan rate of 50 mV s^−1^ in PBS. In DPV, a pulse amplitude of 50 mV, a potential increment of 1 mV and a pulse width of 16.7 ms were employed. EIS spectra of the DNA sensors were recorded in a solution consisting of K_3_Fe(CN)_6_/K_4_Fe(CN)_6_ (0.005M) and 0.1 M KCl at a frequency range from 10^5^ Hz to 0.1 Hz.

The electrochemical method was used to determine the effective surface area (A_eff_) of working electrodes using K_3_[Fe(CN)_6_] as a probe [34]. CV of 5 mM K_3_[Fe(CN)_6_] in 0.1 M KCl at bare electrode was obtained at 50 mVs^−1^ scan rate. The effective surface areas of gold electrodes were calculated according to the previously reported method [35].

The DNA surface coverage (Γ, pmol/cm^2^) was calculated using Equation (1).

(1) $$\Gamma =\frac{Q}{nFA_{eff}}$$

where *Q* (C), *n*, *F*, and *A_eff_* represent the reduced charge of an electrochemical indicator (MB) in cyclic voltammetry obtained for the ssDNA-Au electrodes, the quantity of electrons involved in the reaction (2e^−^), the Faraday constant (96,485 × 10^−12^ C/pmol), and the effective area of the Au disk electrode, respectively [36,37]. The DNA surface coverage was determined by integrating the second scan’s reduction peak of cyclic voltammograms.

## 3. Results and Discussion

### 3.1. Design of the DNA Beacon Sequences

The hairpin beacons were designed using the DNA sequence of one of the most investigated genes in cancer research, the TP53 gene. A 28 nt hairpin DNA strand with a stem and a loop was designed to complement the TP53 biomarker. The complementation sequence detection of the arginine-tryptophan mutation sequence was designed using a single nucleotide polymorphism and the triple base mismatch (TBM) sequence. The detection of single base mismatch (SBM) with a mutation (G to A) at residue R248 enables sensitive recognition to frequently occurring cancer-causing mutations.

### 3.2. Characterization of the Self-Assembled DNA Sensor

**Figure 2:**
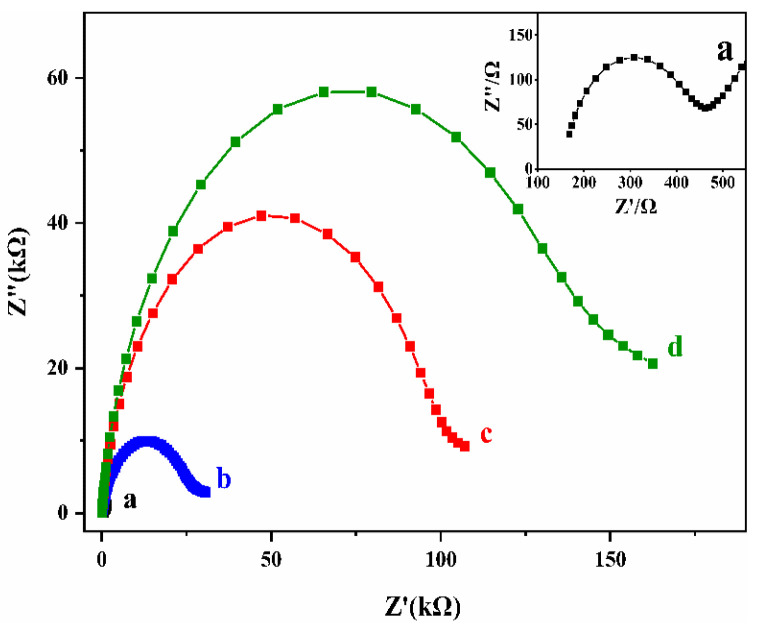
EIS of bare Au electrode (a), ssDNA modified Au electrode (b), MCH modified ssDNA-Au electrode (c), and the biosensor after incubated with cDNA (d).

The DNA beacons were mounted on gold electrodes and electrochemically characterized in their folded and open states (i.e., prior to and following hybridization with the target DNA). Biosensor fabrication and EIS measurements were characterized using 0.1 M KCl containing 5 mM K_3_Fe(CN)_6_ and K_4_Fe(CN)_6_. As shown in Figure 2, the semicircle at a high frequency in EIS, represents the charge transfer process, and its diameter represents the charge transfer impedance (Rct) value. The low-frequency linear part represents the electron transfer process of mass diffusion control [38,39]. In comparison to the bare Au electrode (curve a), the ssDNA-Au electrode demonstrated a significantly higher electron-transfer resistance R_ct_ (curve b), owing to the negatively charged phosphate backbone of the ssDNA bound on the electrode’s surface. It will repel [Fe(CN)_6_]^3−/^^4−^, which also have negative charges. Electron transfer is limited by increased steric hindrance and charge accumulation on the electrode surface [40], resulting in a considerable increase in R_ct_. Subsequent surface blocking with MCH led to a further increase of R_ct_ (curve c), indicating the successful preparation of an electrochemical biosensor. After hybridization of the biosensor with the target cDNA, dsDNA can be formed. On the electrode surface, a huge amount of negatively charged DNA can be accumulated to block electron transmission, resulting in a significantly increased Rct (curve d).

### 3.3. Electron Transfer Dynamics Study in MB-Labeled Electrodes

**Figure 3:**
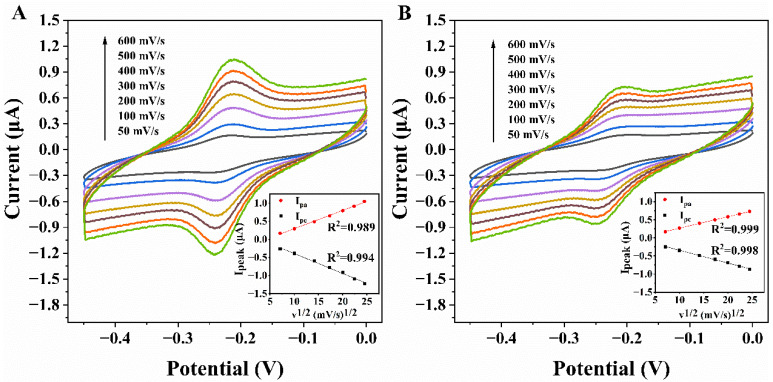
Cyclic voltammograms of ssDNAAu (**A**) and dsDNA-Au (**B**) at different scan rates in pH 7.4 PBS and plot of peak currents vs. square root of scan rates (Inset).

CV was used to determine the dynamics of the response in ssDNA-Au and dsDNA-Au with a scan rate range of 5 to 600 mV s^−1^. In Figure 3A, the peak currents of the anodic and cathodic gradually increase as the scan rates increase. The peak currents are clearly proportional to the square root of the scanning rates (Figure 3A). Upon hybridization, despite the fact that MB is far from the electrode surface due to the rigid structure of the double-stranded DNA, resulting in a low electron transfer (ET) rate and reduced current, the peak currents still have a clear linear relationship with the square root of the scan rates (Figure 3B). They are indicative of a surface-controlled electron transfer process on the electrode’s surface [39,41,42]. Compared to the dsDNA-Au electrode, a more rapid electron transfer rate and larger response current are demonstrated on the ssDNA-Au electrode.

### 3.4. The Feasibility of the DNA Sensor

**Figure 4:**
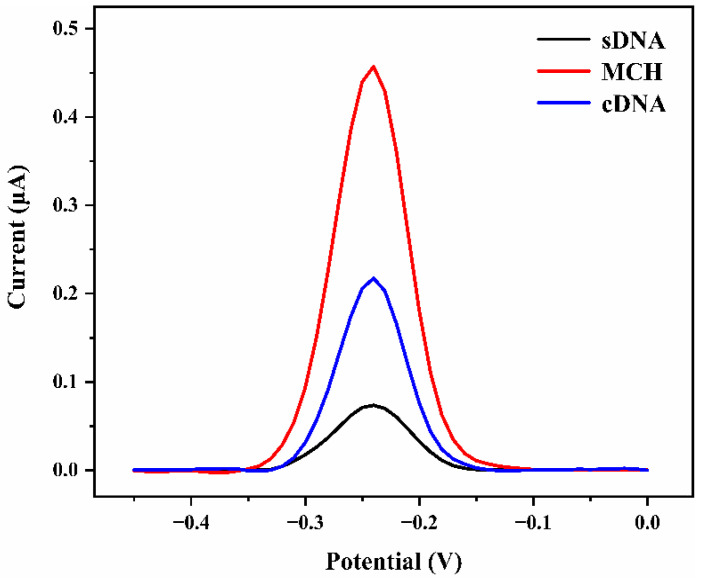
DPV curves of ssDNA-Au electrode, MCH modified ssDNA-Au electrode, and the biosensor after incubated with 1 μM cDNA.

We used DPV to examine the feasibility of the DNA sensor (Figure 4). A well-defined peak of MB was found at −0.25 V when the electrode was coated with ssDNA tagged MB. The oxidation peak of MB rose noticeably when the end-sealing agent MCH was immobilized on the electrode surface. The reason for this is that under the action of MCH, ssDNA that was previously distributed irregularly on the electrode surface can be fixed on the electrode with an upright structure, decreasing the distance between MB and the electrode (Figure 1). The distance between MB and the gold electrode increased after hybridization with target cDNA for 30 min, resulting in a significant decrease in peak current.

### 3.5. Analysis of Impact Factors

By comparing the changes in peak currents measured by CV and DPV, the effect of probe and cDNA concentration on sensor measurement can be determined. The probe concentration, as shown in Figure S1A, is a significant factor that influences sensor performance. 5 µL aliquots of ssDNA solutions at concentrations of 0.01, 0.1, 1, and 10 µM were fixed on the treated gold electrode’s for 16 h at 4 °C and then immersed in MCH for 1 h. Coverages of 0, 2.9, 11.55, and 9.57 pmol/cm^2^ were calculated, respectively (Equation (1)). The maximum theoretical surface coverage (30–45 pmol/cm^2^) is noticeably higher than the reported values for the DNA monolayer [43,44]. Figure S1B shows the probe coverage on the electrode rose first and then decreased, which is in agreement with the change of CV peak current. We applied 1 μM ssDNA for the next studies to maximize the coverage of ssDNA considering the cost of labelled DNA.

**Figure 5:**
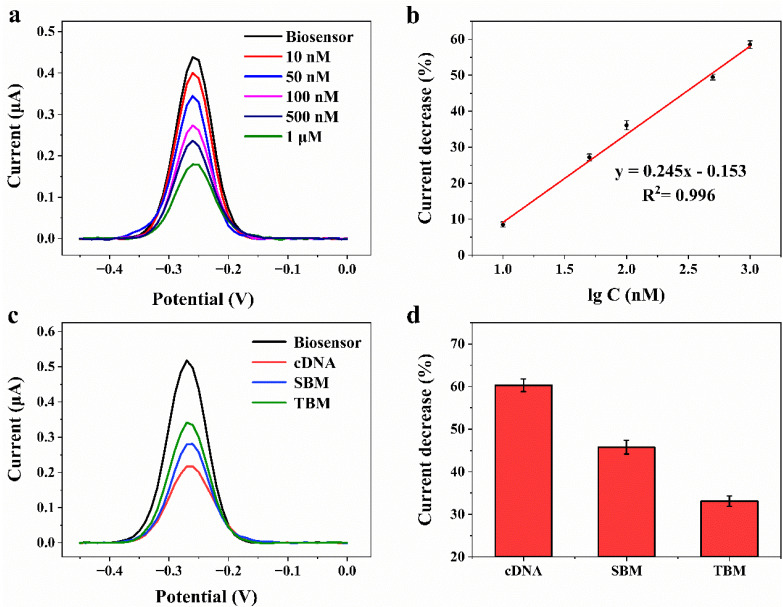
(**a**) DPV curves of the geno-sensor after 30 min hybridization to cDNA at concentrations of 10 nM, 50 nM, 100 nM, 500 nM, and 1 μM in pH 7.4 PBS. (**b**) The peak current decrease percentage vs. the logarithm of cDNA concentrations. (**c**) DPV curves of the electrochemical geno-sensor after 30 min hybridization to 1 μM cDNA, SBM and TBM, respectively. (**d**) Peak current decrease percentage of the geno-sensor upon exposure to 1 μM cDNA, SBM, and TBM, respectively.

The signal changes were studied by hybridization with different concentrations of 21 nt cDNA sequences at the constant probe concentration of 1 μM for 30 min (Figure 5a). The concentrations of 21 nt cDNA were 10 nM, 50 nM, 100 nM, 500 nM, and 1 μM, respectively, and the percentage of peak current decrease in DPV demonstrated a good linear relationship with the logarithm of cDNA concentration (Figure 5b). The detection limit of the electrochemical DNA sensor was up to 10 nM due to the hairpin structure and signal amplification of MB. The obtained detection limit for TP53 is significantly better than that of the field-effect transistor sensor (100 nM) [45], and is comparable to that of the electrochemical biosensor developed by Otero et al. [37]. With an increase in cDNA concentration, the binding efficiency of the double-stranded was improved, and a larger amount of MB was far away from the electrode surface, resulting in a significant decrease in peak current. When the concentration was increased to 1 μM, the percentage of peak current reduction for 21nt-cDNA reached 60%, providing an ideal signal difference for cDNA detection. As can be seen, the DNA sensor showed high specificity and sensitivity for complementary TP53 gene detection. In order to further explore the effect of cDNA sequence length on response signal, the gene sensor was hybridized with 28nt-cDNA under the same conditions. As shown in Figure S2, the percentage of geno-sensor peak current reduction for 21nt-cDNA was much higher than for 28nt-cDNA, which demonstrates that the designed geno-sensor is more sensitive for 21nt-cDNA. So, 21nt-cDNA is preferable for further study.

### 3.6. The Specificity of the DNA Sensor

To further investigate the specificity of the DNA sensor, the SBM and TBM sequences were hybridized with DNA sensors, respectively, as shown in Figure 5c. Compared with the peak current value before hybridization, the change rate of the peak current value $\Delta_{\mathrm{et}}=\left(I_{Biosensor}-I_{dsDNA}\right)\;/\;I_{Biosensor}$ after hybridization with different hybridization sequences is shown in Figure 5d. When the ssDNA sensor hybridizing with 1 μM cDNA, the Δ_et_ value is 60.29 ± 1.48%. When the ssDNA sensor hybridizing with 1 μM TBM or SBM, the Δ_et_ values are 45.55 ± 1.60%, 32.14 ± 1.23%, respectively. As can be seen, the DNA sensor displayed an ideal signal difference for detecting single base mismatches (SNP) and three-base mismatches base sequences. The mutation site of the single-base mismatch sequence is the R248 mutation hotspot of the TP53 gene, and the peak current change percentage of the biosensor for cDNA and SBM is quite different. This demonstrates that the DNA sensor can be used for the early diagnosis or early warning of cancer related to the R248 mutation hotspot of TP53.

### 3.7. The Reproducibility, Repeatability and Stability of the DNA Sensor

**Figure 6:**
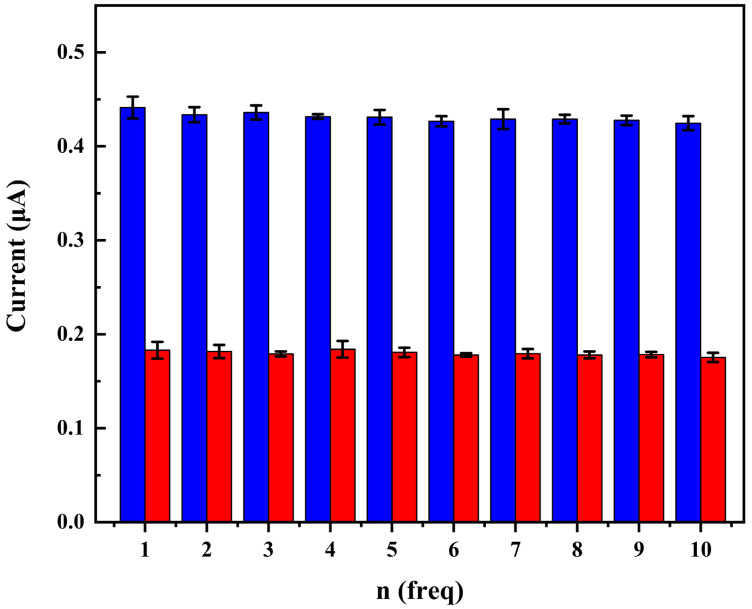
DPV response peak currents of five different ssDNA-Au biosensors as a function of scans from 1 to 10 in pH 7.4 PBS before (■) and following hybridization with 1 μM cDNA (■).

The reproducibility, repeatability, and stability of the DNA sensor were studied by using the DPV technique. The results of 10 consecutive potential scans of the prepared five different biosensors are shown in Figure 6. The relative standard deviation (R.S.D.) of the same biosensor response to 1 μM cDNA for 10 successive measurements was within 1.3%, indicating good repeatability and operational stability. To evaluate the reproducibility between DNA sensors, five biosensors were prepared under the same conditions independently. The R.S.D. of the obtained five gene-sensors was within 2%, indicating good reproducibility for electrode batches.

### 3.8. Detection of Target Molecules in Complex Systems

Early diagnosis of cancer is usually achieved by detecting tumor markers present in tumor tissue, serum, and peripheral blood [46]. However, the serum is a complex mixture, including plasma proteins, peptides, fats, carbohydrates, hormones, and so on. These substances are likely to have a certain impact on the detection signal of biosensor. Due to the fact that 50% fetal bovine serum (FBS) contains similar components to human serum, we used it to verify the anti-interference ability of the developed electrochemical biosensor in the complex detection environment. 50% FBS was used to simulate a complex biological sample for the detection of target DNA molecules, and the detection result was shown in Figure S3. The co-existing FBS did not interfere with the detection of target DNA, and the gene-sensor can still effectively distinguish target molecules with single base mismatch, indicating that the sensor can selectively identify target molecules in complex systems, and could be applied to clinical sample detection.

## 4. Conclusions

In summary, an electrochemical geno-sensor based on hairpin-DNA was developed to detect the mutation hotspot sequence of the TP53 tumor suppressor gene. A highly ordered hairpin DNA monolayer was formed on the surface of the gold electrode by self-assembly method. With the hybridization reaction with the target sequence, the hairpin probe structure was induced to rearrange into linear DNA double chains, and TP53 gene detection at nanomolar concentration was realized. The results demonstrate that the peak current decrease percentage of the sensor has a good linear relationship with the logarithm of the target DNA concentration (R^2^ = 0.998) with a low detection limit of 10 nmol L^−1^. More importantly, the biosensor showed sensitive specific recognition ability for a single nucleotide polymorphism in the TP53 gene affecting residue 248 of the P53 protein. There are about 15% and 28% difference in the change rates of peak currents for SBM and TBM compared to cDNA. At the same time, the gene-sensor demonstrated good reproducibility and stability and excellent anti-interference ability for the target DNA molecule in the complex system of 50% fetal bovine serum. It is expected to be applied to the detection of clinical samples and provides a promising rapid method for the early diagnosis and early detection of cancer.

## Data Availability

Not applicable.

## Supplementary Materials

The following supporting information can be downloaded at: https://www.mdpi.com/article/10.3390/bios12080658/s1, Table S1: The used oligonucleotides in this study; Figure S1: CV of a gold electrode after 16 h exposure to ssDNA at concentrations of 10 nM, 100 nM, 1 μM and 10 μM in pH 7.4 PBS (A), and electrode surface coverage of ssDNA probes with different concentrations (B); Figure S2: DPV curves of the electrochemical geno-sensor after 30 min hybridization to 1 μM 21 nt cDNA and 28 nt cDNA, respectively; Figure S3: Detection capabilities of the DNA sensors for target DNA in complex systems of 50% fetal bovine serum.
