# Supplementary material for: Sensitive Electrochemical Biosensor for Rapid Screening of Tumor Biomarker TP53 Gene Mutation Hotspot
Source: Biosensors (Basel). 2022 Aug 19;12(8):658. doi: 10.3390/bios12080658 (PMC9406039; doi:10.3390/bios12080658)
Supplement: Supplementary file 1 [file biosensors-12-00658-s001.zip › biosensors-1849412-supplementary.pdf]

Supporting Information

Sensitive electrochemical biosensor for rapid screening of tumor biomarker TP53 gene mutation hotspot

Table S1. The used oligonucleotides in this study.

Name	Sequence (5'→3')
ssDNA	HS-SH-$(CH_2)_6$-GGC ATG AAC CGG AGG CCC ATC TCA TGC C-MB
21nt-cDNA	GAT GGG CCT CCG GTT CAT GCC
28nt-cDNA	GGCATGAGATGGGCCTCCGGTTCATGCC'
SBM (single base mismatch)	GAT GGG CCT CCA GTT CAT GCC
TBM (triple base mismatch)	GAT GGG CCT CAA ATT CAT GCC

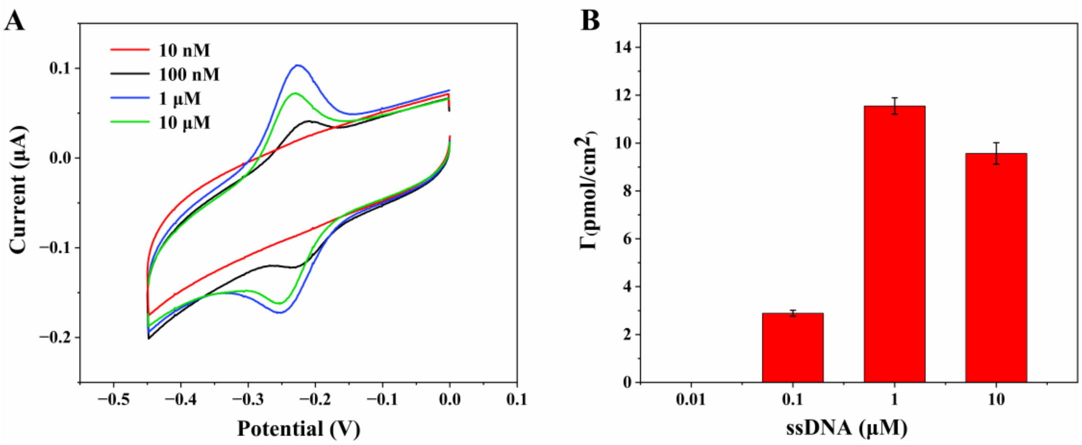

Figure S1. CV of a gold electrode after 16 h exposure to ssDNA at concentrations of 10 nM, 100 nM, 1 μM and 10 μM in pH 7.4 PBS (A), and electrode surface coverage of ssDNA probes with different concentrations (B).

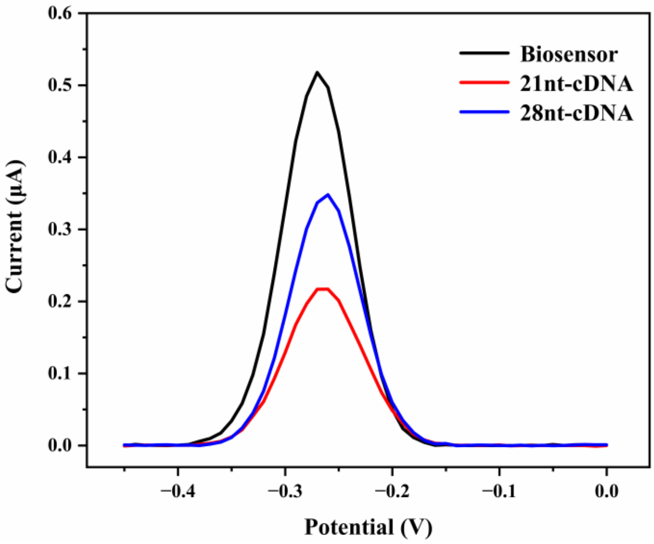

Figure S2. DPV curves of the electrochemical geno-sensor after 30 min hybridization to 1 μM 21 nt cDNA and 28 nt cDNA, respectively.

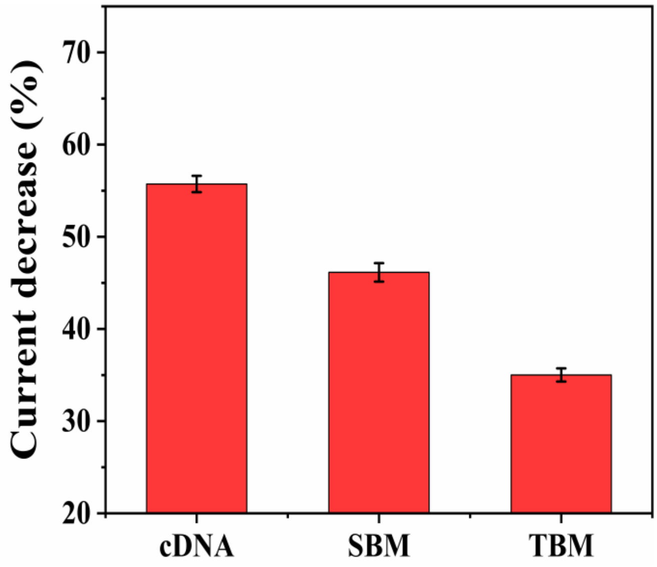

Figure S3. Detection capabilities of the DNA sensors for target DNA in complex systems of 50% fetal bovine serum.
